# Hydrogen Sulfide and *β*-Synuclein Are Involved and Interlinked in the Aging Glaucomatous Retina

**DOI:** 10.1155/2020/8642135

**Published:** 2020-04-13

**Authors:** Hanhan Liu, Karl Mercieca, Fabian Anders, Verena Prokosch

**Affiliations:** ^1^Experimental Ophthalmology, Department of Ophthalmology, University Medical Center of the Johannes Gutenberg University, Mainz 55131, Germany; ^2^Manchester University Hospitals NHS Trust, Manchester Royal Eye Hospital, Manchester M13 9WH, UK; ^3^Faculty of Biology, Medicine and Health School of Health Sciences, University of Manchester, Manchester M13 9WH, UK

## Abstract

**Purpose:**

Glaucoma, one of the leading causes of irreversible blindness worldwide, is a group of disorders characterized by progressive retinal ganglion cell (RGC) loss. Synucleins, a family of small proteins, have been of interest in studies of neurodegeneration and CNS. However, their roles and functions in glaucoma are still not completely understood and remain to be explored. Our previous studies showed that *α*-synuclein and H_2_S play a pivotal role in glaucoma. This study aims to (1) elucidate the potential roles and functions of synucleins in glaucoma throughout aging, (2) investigate the interaction between the synucleins and H_2_S, and better understand the mechanism of H_2_S in neuroprotection.

**Methods:**

The chronic IOP elevation model was carried out in 12 animals at different ages (3 months and 14 months), and RGCs were quantified by Brn3a staining. Mass spectrometric-assisted proteomics analysis was employed to measure synuclein levels and H_2_S producing proteins in retina. Secondly, the acute IOP elevation model was carried out in 12 juvenile animals, with or without intravitreal injection of GYY4137 (a H_2_S donor). RGCs were quantified along with the abundancy of synucleins.

**Results:**

RGCs and *β*-synuclein (SNCB) are significantly changed in old animals. Under chronic IOP elevation, there is a significant RGC loss in old animals, whereas no significant change in young animals; SNCB is significantly downregulated and 3MST is significantly upregulated in young animals due to IOP, while no significant changes in old ones are notable. Under acute IOP elevation (approx. 55 mmHg), a significant RGC loss is observed; exogenous H_2_S significantly reduced RGC loss and downregulated SNCB levels.

**Conclusion:**

The present study indicates a strong link between ageing and SNCB regulation. In young animals SNCB is downregulated going along with less RGC loss. Furthermore, increasing endogenous H_2_S is effective to downregulate SNCB and is neuroprotective against acute IOP elevation.

## 1. Introduction

Glaucoma, one of the leading causes of irreversible blindness worldwide [1], is a group of disorders characterized by progressive retinal ganglion cell (RGC) loss and axon atrophy, which leads to gradually visual field loss [2]. By far the only known modifiable risk factor of glaucoma is intraocular pressure (IOP); however, lowering IOP is not able to halt the deterioration of glaucoma in most patients in clinic practice, indicating again the multifactorial pathogenesis and the complexity of glaucoma [3]. The other main risk factor is age. Alternative approaches independent of IOP and probably combating aging as well as focusing on the pathophysiological processes are in demand to ameliorate glaucoma neuropathy. Other pathophysiological processes including oxidative stress, inflammatory reaction, glial activation, vascular dysfunctions, and abnormal protein accumulation are proven to be closely involved [4–7].

Hydrogen sulfide (H_2_S) has been recognized as the third endogenous gaseous signaling molecule alongside carbon monoxide (CO) and nitric oxide (NO) [8]. As a potent reductant, H_2_S plays critical roles in multiple physiological and pathological processes, it works to alleviate inflammatory responses and oxidative stress and restores energy shortage [9–11]. H_2_S has shown profound therapeutic efficiency potential in neurodegenerative diseases in CNS [12–15]. Research studies focused on H_2_S in connection with glaucoma have increasingly emerged in last few years; our knowledge on this topic is still lacking and remains to be thoroughly expanded. Alteration of endogenous H_2_S level in retina is correlated with different pathological situations, and its exogenous donors exhibited potential in protecting retinal ganglion cells against assaults, such as diabetic retinopathy, ischemia-reperfusion injury, and *N*-methyl-D-aspartic acid- (NMDA-) induced excitatory neurotoxicity [16–18]. In our previous study, alteration of endogenous H_2_S synthases is observed in a glaucoma animal model; furthermore, we observed that GYY4137, a slow-release H_2_S donor, effectively protected RGCs against different glaucomatous injuries in vitro and in vivo [19]. The neuroprotective effect of H_2_S was partly attributed to its capability of vasorelaxation, antioxidative stress, neuroendocrine regulation, and inflammation suppression [20–22], but the internal mechanism underlying it is still unclear.

Synuclein is a family of small proteins including *α* (SNCA), *β* (SNCB), and *γ* (SNCG) synucleins [23, 24] and is involved in various neurodegenerations in the CNS. Specifically, SNCA is a major constituent of Lewy bodies (LB) and pathological neuronal inclusion bodies found in Parkinson's disease (PD), Alzheimer's disease (AD), and other neurodegenerative disorders [25, 26]. Mutations of SNCA play a central role in PD pathology, and misfolding and aggregation of SNCA directly linked to microglial activation, followed by inflammation and oxidative stress resulting in neurodegeneration [27]. Synucleins are present in the retina and optic nerve [28] and are associated with glaucomatous alterations in the optic nerve [29]. SNCA autoantibody was found to be downregulated in serum and upregulated in aqueous humor of glaucoma patients [30], and in our previous study, intravitreal injection of SNCA antibodies is found to be neuroprotective in a glaucoma animal model [31].

*β*-Synuclein shares a similar protein structure to SNCA [32], but lacks the nonamyloid-*β* component domain [33]. Its expression is documented to be increased in cerebrospinal fluid in patients with neurodegenerative diseases and in neuronal retina and visual cortex of rats and nonhuman primates with age and external stress [34–36]. SNCB is thought to function as a physiological inhibitor of SNCA in neurodegenerative diseases [36, 37], and it retains antiapoptotic ability in a dose-dependent manner [23], and *β*-synuclein-derived peptides behave as antiaggregating agents [25]_ENREF_7.

While SNCA and SNCB are mainly associated with diseases in the CNS, *γ*-synuclein is first identified as breast-specific gene protein 1 [38], but it is also involved in axonal spheroid-like lesions in Parkinson's disease, deposition in glial cells in glaucoma, and motor neuron dysfunction and death [29, 39, 40].

Because of the pivotal role of SNCA in neurodegeneration in CNS, it has been extensively studied in CNS. But its role in retina or glaucoma, as well as SNCB and SNCG's roles and functions in glaucoma is still sparse and remain to be thoroughly explored.

Studies have shown that H_2_S and synucleins are involved in several mutual pathophysiological processes, such as microglia activation, p53-mediated apoptosis, inflammatory response, and free radical reactions [8, 11, 41, 42]. Purpose of this study is to first elucidate the potential roles and functions of synucleins in glaucomatous neuropathy, following this, to investigate the potential of H_2_S to regulate it and to better understanding the mechanism of H_2_S in neuroprotection.

## 2. Method

### 2.1. Animal Treatment and Ethical Statement

Female Sprague-Dawley rats (*n* = 24,250–300 g) were used for this study. All experimental procedures were conducted in accordance with the Association of Research in Vision and Ophthalmology (ARVO) Statement for the Use of Animals in Ophthalmic and Vision Research and the guidelines of the Institutional Animal Care and Use Committee. The use of animals for research purposes was approved by the Health Investigation Office Rhineland-Palatinate (permission number: 14-1-085; approvals date: 13 October 2014). All animals were housed at the Translational Animal Research Center (TARC) of the University Medical Center of Johannes Gutenberg University Mainz. Food and water were provided ad libitum with a day- and night-circle of 12 hours, respectively.

During experimental interventions, it was prioritized to minimize the discomfort and pain from the animals. For anesthesia, a mixture of medetomidine hydrochloride (Dorbene vet., Pfizer, New York, NY, USA) and Ketamine (Inresa Arzneimittel, Freiburg, Germany) was administered intraperitoneally, and oxybuprocain (Novesine, OmniVision, Puchheim, Germany) was applied topically onto the ocular surface. Novaminsulfon (Novalgin, Ratiopharm, Ulm, Germany) was injected subcutaneously and added to drinking water after the operation to reduce postoperation pain. All animals were observed directly after each intervention and following daily by TARC staff, in terms of their health condition and general behavior.

### 2.2. Study Design and Induction of Glaucoma Models

#### 2.2.1. Induction of Chronic IOP Elevation

The chronic IOP elevation model was carried out in 12 Sprague-Dawley rats at different ages (3 months and 14 months), through episcleral vein occlusion (EVO), described in our previous work, to create a constant IOP elevation for a period of seven weeks [19]. Only left eyes were operated. Briefly, the animals were anesthetized as described above; connective tissue of the eye was carefully opened and the three of the five episcleral vein trunks were cauterized using a medical cauterization device (Bovie Medical Corporation, USA). The IOP was measured before the EVO and on a weekly basis afterwards.

IOP measurement was achieved using a rodent-customized rebound Tonolab (iCare, Finland). Measurements were carried out between 9am and 11am, animals were awake and gently fixated through hand-holding during the measurement. Per eye, ten IOP readings were taken per measurement and subsequently averaged. Animals with fluctuating IOPs or no signs of IOP elevation as a result of the EVO were excluded from the study.

#### 2.2.2. Induction of Acute IOP Elevation

The retinal ischemia-reperfusion injury model is a well-known animal model to mimic clinical manifestations such as retinal vascular occlusion diseases and acute glaucoma and has been widely used for studying retinal neuronal cell damage after ischemic injury. Acute IOP elevation was induced in 12 animals. The animals were anesthetized as described above; before the intervention, 3 *μ*l of saline or GYY4137 (Sigma-Aldrich; Darmstadt, Germany), a slow-releasing H_2_S donor, was injected posterior to the pars plana into the vitreous with a Hamilton syringe (Sigma-Aldrich; Darmstadt, Germany) and 33-gauge needle, the injection volume was 3 *μ*l for optimal distribution of the compound [43]. To avoid injection reflux, the needle was kept intravitreal for a period of 15 s. Assuming the vitreous volume of an adult rat eye to be approximately 56 *μ*l [43], the final intraocular concentration of GYY4137 was approximately 100 nM.

Immediately after that, an anterior chamber was carefully cannulated from the superotemporal cornea with a 30-gauge infusion needle; care was taken not to injure the central cornea, lens, and other surrounding tissue. The needle was connected to a plastic container of sterile saline solution. IOP was raised to 55 mmHg by elevating the saline container for 60 min; IOP measurement was conducted by rebound Tonolab (iCare, Vantaa, Finland), designed for rodents. During the procedure, the iris turned pale and the retina lost its red reflex, thus confirmed the ischemia condition. Retinae from untouched contralateral eyes were recruited as baseline control. Animals were euthanized 24 h after the reperfusion injury.

### 2.3. Preparation of Retinal Explants and Quantification of Retinal Ganglion Cells

Sprague-Dawley rats were euthanized under CO_2_ atmosphere. Eyes were enucleated immediately postmortem, and retinae were explanted and flat-mounted as previously described [19]. Briefly, retinal explants were flat-mounted with the ganglion cell side up on millipore filters (Millipore, Millicell, Cork, Ireland), and the vitreous body was removed.

One quarter of each treated retinal explant was carefully separated under the microscope for subsequent immunohistochemical staining against the brain-specific homeobox/POU domain protein 3A (Brn3a). Brn3a immunodetection is a powerful tool to assess RGC survival in several mouse and rat injury models, as shown by Nadal-Nicolas et al. [44, 45]. In brief, retinal tissue was fixed in 4% formalin solution for 30 min (Carl Roth, Karlsruhe, Germany), transferred in 30% sucrose solution, and finally frozen in methylbutane for 10 seconds (Merck, Germany) and then stained subsequently as previously described [46]. Immunofluorescent RGCs were further visualized with a fluorescent microscope (Carl Zeiss, Ltd., Hertfordshire, UK), images were taken with a magnification of 20-fold, 16 images were captured from each quarter of retinal flat-mounts, and great effort was taken to ensure the proportion of central and peripheral retinal images was identical in every individual retinal piece. Total numbers of Brn3a positive cells were counted with the assistance of ImageJ (ImageJ Fiji v_1).

### 2.4. Optical Coherence Tomography

Spectral domain-optic coherence tomography (SD-OCT) (Heidelberg Engineering, Germany) was employed in this study to measure the thickness of the retinal nerve fiber layer (RNFL), inner nuclear layer (INL), and outer nuclear layer (ONL). For all measurements, eye-track and progression analysis was applied. Optic nerve head was centralized in the fundus picture; 100 frames of 12-degree circular B-scan were captured and subsequently overlaid. The thickness of different retinal segments was analyzed from the OCT B-scan with the assistance of Heidelberg Eye Explorer software. Manual adjustments were applied to ensure a correct representation of different retinal segments in the software. Baseline OCT measurement was taken before surgical intervention and the final measurement was taken 7 weeks after chronic IOP elevation.

### 2.5. Mass Spectrometry

Remaining retinal tissues of all animals were further utilized for proteomic investigations using an electron spray ionization LTQ Orbitrap mass spectrometer (Thermo Fisher, USA) with an upstream connected liquid chromatography device (LC-ESI-MS).

#### 2.5.1. Sample Preparation

Retina samples were rinsed in ice-cold PBS to remove blood contaminants and weighed; subsequently, the samples were lysed by T-PER Tissue Protein Extraction Reagent (Thermo Scientific Inc., Waltham, MA, USA) and BBY24 M Bullet Blender Storm (Next Advance Inc., Averill Park, NY, USA). According to the manufacturer's instruction, 100 *μ*l T-PER reagent was added to the remaining retinal sample; subsequently, the sample was homogenized by the Bullet Blender Storm and centrifuged at 3,000 ×g for 10 minutes.

The supernatant was collected and pooled together and subsequently cleaned with the Amicon Ultra 0.5 mL centrifugal filters with 3K cutoff (Merck Millipore, Carrigtwohill, Ireland). The protein concentration for each eluate was determined with BCA Protein Assay Kit (Pierce, Rockford, IL). From each sample, 50 *μ*g of the total protein mixture was transferred into 1 × LDS sample buffer (NuPAGE, Thermo Fisher) and subsequently put under reducing condition heated at 80°C for 15 min and separated on a 8% Bis-Tris gel ((Invitrogen, Karlsruhe, Germany) for 30 minutes at 180 V in 1 × MES buffer. SeeBlue Plus 2 (Invitrogen, Karlsruhe, Germany) was used as a molecular mass marker. Colloidal Blue Staining Kit (Invitrogen, Karlsruhe, Germany) was used to stain the gel. Protein lanes were sliced into 20 bands per replica and cut into small pieces and destained with the mixture composed of 1 : 2 (vol/vol) of 100 mM ammonium bicarbonate (NH_4_HCO_3_) and acetonitrile, accordingly 10 mM 1,4-dithiothreitol (DTT) in 100 mM ammonium bicarbonate was employed to disulfide bonds and 55 mM iodoacetamide (IAA) in 100 mM NH_4_HCO_3_ for alkylation. Pure acetonitrile was utilized for gel dehydration prior to digesting with sequence grade-modified trypsin (Promega, Madison, USA) in 10 mM NH_4_HCO_3_ and 10% acetonitrile at 37°C overnight. The tryptic peptides were firstly extracted with acetonitrile and then incubated for 30 min at 37°C with a mixture of 5% formic acid and acetonitrile 1 : 2 (vol/vol); the supernatant was pooled and dried in SpeedVac (Eppendorf, Darmstadt, Germany). SOLA SPE plates and cartridges (Thermo Scientific Inc., Waltham, MA, USA) were utilized for further purification of the peptides following manufacture's instruction. The eluate was dried in SpeedVac and stored at −20°C.

#### 2.5.2. Liquid Chromatography- (LC-) Electrospray Ionization- (ESI-) MS/MS

The LC-ESI-LTQ-Orbitrap MS system in our laboratory is well established and optimized to improve sequence coverage and reduce ion suppression effects, details were described in our previous studies [47, 48]. The LC system contains a Rheos Allegro pump (Thermo scientific, Rockford, USA) paired with an HTS PAL autosampler (CTC Analytics AG, Zwingen, Switzerland). The system encompassed a 30 × 0.5 mm BioBasic C18 precolumn (Thermo Scientific, Rockford, USA) connected to a 150 × 0.5 mm BioBasic C18 column (Thermo Scientific, Rockford, USA); the C18 is the hydrophobic alkyl chain which has reversible hydrophobic interactions with the peptides. The reverse phase aqueous solvent A is made of LC-MS grade water with 0.1% (v/v) formic acid, and the organic solvent B is made of LC-MS grade acetonitrile with 0.1% (v/v) formic acid. The gradient had a running time of 90 minutes per gel band as follows: 0–50 min, 10–35% B; 50–70 min, 35–55% B; 70–75 min, 55–90% B; 75–80 min, 90% B; 80–83 min, 90–10% B; and 83–90 min, 10% B [47, 49]. An ESI-LTQ Orbitrap XL-MS system (Thermo Scientific, Bremen, Germany) collects the continuum MS data [50]. The general parameters of the instrument were set as described in detail previously [50]. In brief, positive ion electrospray ionization mode is employed with a spray voltage of 2.15 kV and a heated capillary temperature of 220°C. Data were acquired in an automatic dependent mode switching between Orbitrap-MS and LTQ MS/MS. The Orbitrap resolution was 30000 at *m*/*z* 400 with survey full scan MS spectra. Target automatic gain control (AGC) was set at 1.0 × 10^6^ ions. Polydimethylcyclosiloxane (PCM) at *m*/*z* 445.120025 ions in real time is utilized for internal recalibration and the lock mass option was enabled in MS mode [51]. Top five most intense precursor ions were selected and obtained as tandem data and further subjected for fragmentation by collision-induced dissociation (CID). The normalized collision energy (NCE) was set to 35% with activation time of 30 ms with repeat count of 3 and dynamic exclusion duration of 600 s. The resulting fragmented ions were recorded in the LTQ.

Obtained raw files were analyzed by MaxQuant v.1.5.3.30 (Max-Planck-Gesellschaft, Germany). Parameters were set to a false discovery rate of 0.01 with a minimum peptide length of six. Only unique peptides accounted for the follow-up label-free quantification process. Retinal samples of chronic IOP elevation model were measured individually, and those from acute IOP elevation model were pooled into three biological replicates for the measurement.

### 2.6. Statistics

In all experiments, the control data derived from the contralateral eyes of the respective experimental group did not show any signs of IOP elevation during the whole study. All obtained data regarding are presented as mean ± SD values unless otherwise stated. The averaged RGC density of retinal whole-mounts was calculated per mm^2^. All data were analyzed statistically using grouped parametric *t*-tests for Gaussian distributions, if not, Mann–Whitney *U* testing was used. All statistical calculations and display of the figures were carried out using Prism 8 software (GraphPad Software, Inc., San Diego, CA, USA).

## 3. Results

### 3.1. Chronic IOP Elevation over 7 Weeks due to EVO

**Figure 1:**
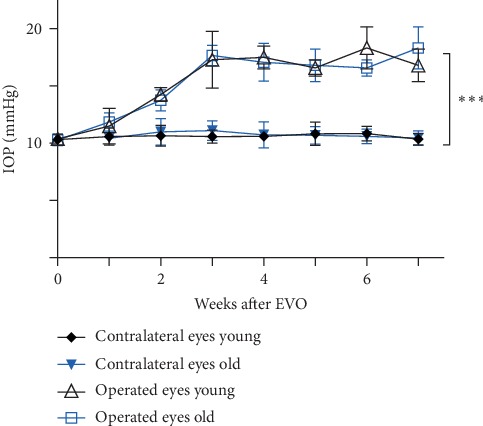
Overview of IOP after episcleral vein occlution for 7 weeks. The intraocular pressure (IOP) of all operated eyes increased significantly (^*∗∗∗*^*p* < 0.001, *n* = 12, mean ± SD) three weeks after the intervention. No noticeable difference in IOP was observed between age groups. The untreated contralateral eyes remained unaffected in terms of IOP changes.

The intraocular pressure (IOP) of all operated eyes increased significantly three weeks after the episcleral vein occlusion. No noticeable difference in IOP was observed between age groups. The untreated contralateral eyes remained unaffected in terms of IOP changes [52] (see Figure 1) (^*∗∗∗*^*p* < 0.001, *n* = 12, mean ± SD).

### 3.2. RGC Density Decreases and Its Susceptibility to Elevated IOP Increases with Age

**Figure 2:**
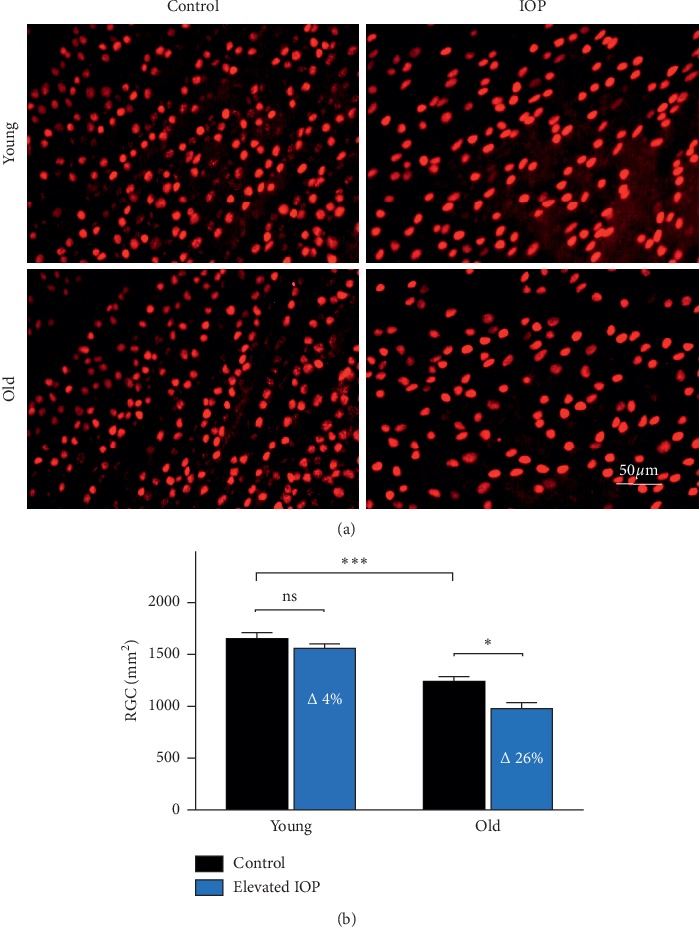
RGC quantification in animals at different ages with or without chronic elevated IOP. (a) Representative fluorescence microscopy of Brn3a staining of retina explants from animals at different ages, with or without elevated IOP. The number of RGCs correlates with the age of the animals and IOP elevation. (b) Independent from IOP, a significant decrease of RGCs could be observed between young and old animals. Elevation of the IOP for 7 weeks leads to a 26% loss of RGCs in old animals, while nonsignificant 4% loss in young animals (^*∗∗∗*^*p* < 0.001, ^*∗*^*p* < 0.05, *n* = 12, mean ± SEM).

A significant decrease in RGC density can be observed between young (3 months old, 1655 ± 57.08 RGC/mm^2^) and old animals (14 months old, 1243 ± 43.58 RGC/mm^2^, ^*∗∗∗*^*p* < 0.001). The chronic IOP elevation over a period of 7 weeks resulted in significant RGC loss in old animals with a reduction of 26% (^*∗*^*p* < 0.05), while in young animals only 4% of RGC loss is observed (see Figure 2) [52].

### 3.3. Morphological Alterations in Retina due to Aging and Chronic Elevated IOP

**Figure 3:**
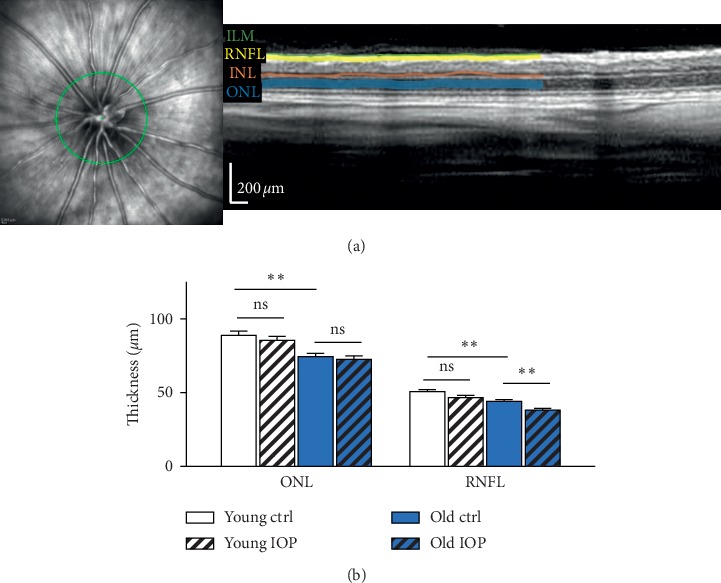
Decrease of the retinal layer thickness as a result of aging and elevated IOP. (a) Exemplary display of the fundus pictures with optic nerve head in the center, and on the right is the OCT B-scan of different retinal layers, internal limiting membrane (ILM), retinal nerve fiber layer (RNFL), inner nuclear layer (INL), and outer nuclear layer (ONL). (b) Compared to young animals, significant thinning is observed in both ONL and RNFL in animals. Elevated IOP only resulted in significant thinning of RNFL while ONL remained unaffected (^*∗∗*^*p* < 0.01, *n* = 12, mean ± SEM).

Measurement of the retinal nerve fiber layer thickness showed comparable results to the RGC density quantification. The thinning of the RNFL was about 8% in young animals and 13% in old animals. Furthermore, an age-related thinning of the RNFL and ONL could be observed; the decrease was about 16% and 13%, respectively (^*∗∗*^*p* < 0.01, *n* = 12, mean ± SEM) (see Figure 3).

### 3.4. Alteration of Endogenous H_2_S in Glaucoma Model

**Figure 4:**
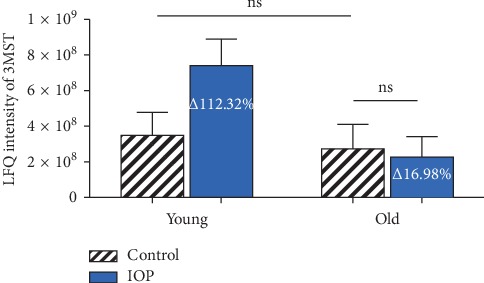
LFQ intensity of 3-mercaptopyruvate sulfurtransferase in retina from animals at different ages with or without chronic elevated IOP. Elevation of the IOP for 7 weeks leads to over 2-fold upregulation of 3MST in young animals, while nonsignificant alteration in old animals. No significant alteration is observed between young and old animals.

In mammalian cells, the endogenous H_2_S is generated on three major pathways: cystathionine-*γ*-synthase (CSE), cystathionine-*β*-lyase (CBS), and 3-mercapto-methylthio pyruvate aminotransferase (3MST). 3MST pathway is the dominating way to produce H_2_S in mammalian retina as 3MST is located in the retinal neurons [53]. In this study, 3-mercaptopyruvate sulfurtransferase in retina is over 2-fold upregulated in young animals due to chronic IOP elevation, while nonsignificant alteration in old animals is visible. There is no significant alteration in expression due to aging between groups (see Figure 4).

### 3.5. Acute Elevated IOP (I/R) Induced Significant Cell Loss in Juvenile Animals, while H_2_S Treatment Protected RGC

**Figure 5:**
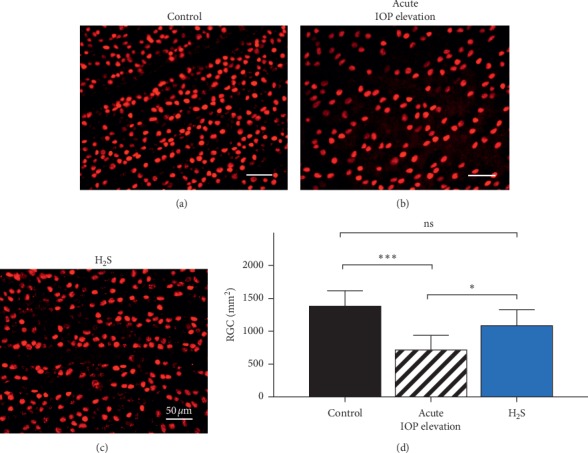
Effect of exogenous H_2_S on RGC loss due to acute IOP elevation. (a–c) Representative fluorescence microscopy of Brn3a staining of retinal explants 24 hours after inducing acute IOP elevation in vivo. (a) Contralateral eye as control. (b) Acute IOP elevation. (c) Acute IOP elevation+100 nM GYY4137. (d) Compared with the contralateral control (1382.8 ± 235.4 RGC/mm^2^), 60 min of acute IOP elevation resulted in significant RGC loss in the experimental eye (714.3 ± 223.4 RGC/mm^2^); pretreatment with GYY4137 significantly reduced RGC loss (^*∗∗∗*^*p* < 0.0005, ^*∗*^*p* < 0.05, *n* = 12, means ± SD).

Compared with the contralateral control (1382.8 ± 235.4 RGC/mm^2^), 60 min of acute IOP elevation resulted in significant RGC loss in the experimental eye (714.3 ± 223.4 RGC/mm^2^); pretreatment with 100 nM GYY4137 significantly reduced RGC loss (1083.5 ± 243.1 RGC/mm^2^). There was no significant difference in RGC numbers between control and H_2_S-treated group, as RGCs were preserved (see Figure 5) (^*∗∗∗*^*p* < 0.0005, ^*∗*^*p* < 0.05, *n* = 12, means ± SD).

### 3.6. Altered Synuclein Levels in Acute Elevated IOP Model with or without H_2_S Treatment

**Figure 6:**
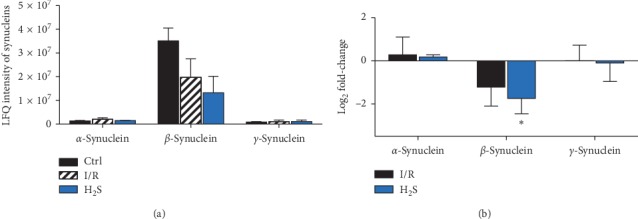
LFQ intensity and alteration of synucleins in the I/R animal model with or without H_2_S treatment. (a) LFQ intensity of synucleins following LC-ESI-LTQ-Orbitrap mass spectrometry. (b) Acute elevated IOP has the tendency to downregulate the *β*-synuclein (not statistically significant), while administration of H_2_S significantly reduced the abundancy of *β*-synuclein (FC = −1.749, ^*∗*^*p* < 0.05, *n* = 6, mean ± SEM). Either I/R injury or H_2_S has significant impact on *α*-/*γ*-synuclein.

According to label-free quantification process following LC-ESI-LTQ-Orbitrap mass spectrometry, acute elevated IOP has the tendency to downregulate the *β*-synuclein (not statistically significant), while administration of H_2_S significantly reduced the abundancy of *β*-synuclein (FC = −1.749, ^*∗*^*p* < 0.05, *n* = 6, mean ± SEM). Either I/R injury or H_2_S has significant impact on *α*-/*γ*-synuclein (see Figure 6).

### 3.7. Altered Synuclein Levels in Animals at Different Ages with or without Chronic Elevated IOP

**Figure 7:**
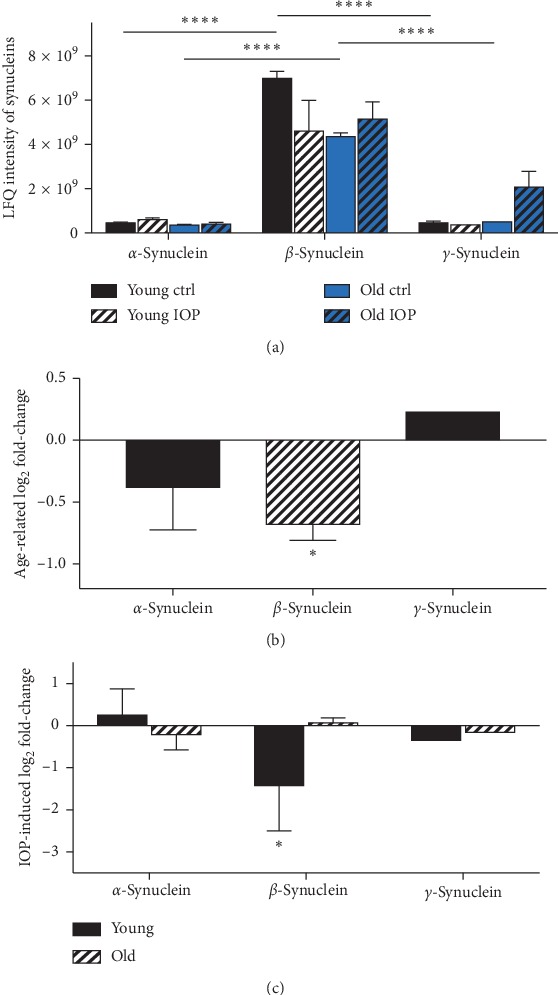
LFQ intensity and alteration of synucleins in retina from animals at different ages with or without chronic elevated IOP. (a) LFQ intensity of synucleins following LC-ESI-LTQ-Orbitrap mass spectrometry. In both young and old animals, *β*-synuclein is significantly more abundant than *α*-synuclein and *γ*-synuclein in retina. (b) Independent from IOP, *β*-synuclein is significantly downregulated due to aging, while nonsignificant alteration in *α*-/*γ*-synuclein is observed. (c) Elevation of the IOP for 7 weeks leads to significant downregulation of *β*-synuclein in young animals, while nonsignificant change in old animals. No significant alteration of *α*-/*γ*-synuclein is observed in both groups (^*∗∗∗∗*^*p* < 0.001,^*∗*^*p* < 0.05, *n* = 12, mean ± SEM).

Label-free quantification process following LC-ESI-LTQ-Orbitrap mass spectrometry shows that *β*-synuclein is significantly more abundant than *α*-/*γ*-synuclein in retina in both young and old animals. Furthermore, a significant downregulation of *β*-synuclein (FC = −0.679) is observed with aging, while nonsignificant age-related alteration in *α*-/*γ*-synuclein. The chronic IOP elevation over a period of 7 weeks resulted in significant downregulation of *β*-synuclein (FC = −−1.428) in young animals, while nonsignificant change in old animals. Elevated IOP does not show significant impact on *α*-/*γ*-synuclein in both animal groups (see Figure 7) (^*∗∗∗∗*^*p* < 0.0005, ^*∗*^*p* < 0.05, *n* = 12, mean ± SEM).

## 4. Discussion

In our previous work, we demonstrated that exogenous H_2_S supplement and *α*-synuclein antibodies significantly improved the RGC survival in different experimental glaucoma [19, 31]. However, the underlying mechanisms remained to be explored. In this study, we investigated the level changes of synucleins in the retina in different glaucoma animal models, a chronic progressive model of glaucoma at different age stages and an acute IOP elevation induced by ischemia-reperfusion injury. Furthermore, as synucleins and H_2_S are involved in several mutual pathophysiological processes, we expect to explore the correlation between H_2_S and synucleins in order to better understand the mechanism H_2_S's neuroprotective properties in experimental glaucoma.

In this study, we had the following findings:

1. In rat retina, SNCB has significantly higher abundancy than *α*-/*γ*-synuclein
2. SNCB decreases with age in rat retina
3. In respond to chronic elevated IOP, SNCB is significantly downregulated in juvenile animals while no significant change is observed in old animals
4. Downregulation of SNCB and upregulation of H_2_S is correlated with reduced cell loss due to chronic elevated IOP in juvenile animals
5. Exogenous H_2_S significantly reduced cell loss due to acute elevated IOP
6. Exogenous H_2_S significantly downregulates SNCB in rat retina

In first part of this study, glaucoma was mimicked through mildly elevated IOP for seven weeks. Subsequent loss of RGCs is in agreement with thinning of the retinal nerve fiber layer, which suggests that EVO is a sufficient glaucoma model. As predominantly elder people are affected in glaucoma, older animals showed a higher susceptibility to IOP elevation resulting in significant loss of RGCs and RNFL thickness, while younger animals seemed to show resistance against mildly elevated IOP.

Comparing to young animals, thickness of both ONL and RNFL is decreased considerably in old animals. It has been shown that the cyclic light intensities under which the rats are reared have impacts on the rod outer segment length and photoreceptor cell density [54]. Especially in albino animals such as Sprague-Dawley rats, the retina of caged rodents develops light damage in old age. Therefore, thinning in ONL and RNFL indicates an overall decrease of retinal neurons, the observed chronic RGC decline in aged animals is likely due to chronic light damage.

Secondly, we employed label-free quantification process following LC-ESI-LTQ-Orbitrap mass spectrometry to measure the abundance of synucleins and 3MST in the retina. We found that SNCB has significantly more abundant retina than other family members; furthermore, its level is significantly altered due to aging and elevated IOP, while the other two family members did not show noticeable changes, which indicates that SNCB might have a more pivotal role to play in neurodegenerations in retina than other family members.

In physiological aging, the abundance of SNCB declines, which is correlated with the decreased RGC density and increased susceptibility to IOP. As under physiological conditions, SNCB is thought to be neuroprotective by functioning as a physiological inhibitor of SNCA and behaving as antiaggregating agents. Studies in autopsy brains of PD, dementia with Lewy bodies, and AD suggest that decreased amount of SNCB may lead to relative loss of protective functions of SNCB against neurotoxicity caused by SNCA [55]. Furthermore, downregulation of SNCB could occur not only in aggregation of SNCA, but also in other types of neurodegenerative disease [56].

SNCB's downregulation with aging increases RGC's susceptibility to glaucomatous assaults secondary to elevated IOP, such as elevated mechanic stress, insufficient retinal perfusion, and increased oxidative stress.

Under pathological conditions, chronic elevated IOP, SNCB in juvenile animals is downregulated, and the downregulation of SNCB is correlated with reduced RGC loss. While in aged animals, there is no significant alteration of SNCB in response to assault, but more significant RGC loss.

According to mass spectrometry results, the amount of 3MST in rat retina is not significantly altered through the process of aging. When exposed to elevated IOP over a period of 7 weeks, 3MST was significantly upregulated in juvenile animals, while no significant change is observed in old animals. Associating with the data from immunofluorescence staining of RGC, it suggests that upregulation of 3MST, a key H_2_S-producing enzyme, is correlated with reduced RGC loss induced by elevated IOP. The self-regulation of H_2_S is decreased with aging. Therefore, we assume that downregulating SNCB and upregulating endogenous H_2_S level are neuroprotective against elevated IOP, and the function of regulating them is weakened with aging, which renders RGC's vulnerability.

Furthermore, acute IOP elevation is induced in juvenile animals, which led to significant RGC loss and SNCB downregulation. Although juvenile animals are more resilient than old animals to mildly elevated IOP (ca. 18 mmHg), but when it reaches a threshold, acute IOP elevation (IOP ca. 55 mmHg) led to a significant RGC loss. Downregulation of SNCB might be therefore a self-protective mechanism presenting from the beginning of the IOP elevation, but an exhaustion of the functional reserve eventually led to RGC loss.

Our data on SNCB abundance and RGC loss agree with recent studies in retina, showing that the protective property of SNCB is exerted in a dose-dependent manner [23], which means overexpression and accumulation of SNCB increase oxidative stress and inflammatory responses, and furthermore promote the apoptosis, while lower concentrations of SNCB show antiapoptotic effect [36, 57]. In various aspects of neurodegeneration, accumulation of SNCB is present, such as in dystrophic neurites in the hippocampal region in brains from PD and DLB patients, which suggest that accumulation of SNCB is involved in the axonal pathology [39]. SNCB was found to form toxic cytosolic inclusions in a similar manner to SNCA and shares similar toxicity mechanisms, including vesicular trafficking impairment and induction of oxidative stress [58]. Overexpression of SNCB in cultured primary cortical neurons led to cell loss and signs of metabolic impairment, in a similar manner to overexpressing SNCA neurons [59].

Treatments targeting SNCA to reduce its levels and toxicity have shown positive results in rescuing neuronal cells and halting the neurodegeneration process in preclinical studies [56, 60]. For example, in our previous study, intravitreal injection of SNCA antibodies is found to be neuroprotective in a glaucoma animal model [31].

Thus, it is reasonable to target the pathogenic SNCB and to decrease the intracellular SNCB as novel strategies for therapeutic intervention in neurodegeneration. Removal of pathogenic SNCB or to reduce its abundancy may be effective to rescue neuron and halt the progression of glaucoma.

H_2_S has shown profound involvement in various retinal neuropathy processes; in previous studies by different groups including us, exogenous donors exhibited therapeutic potential in conditions of several retinal diseases [11, 19]. The underlying mechanism, through which H_2_S exerts its neuroprotection, was partly attributed to its capability of vasorelaxation, antioxidative stress, neuroendocrine regulation, and inflammation suppression [20–22]. Moreover, H_2_S is involved in several mutual pathophysiological processes with SNCB, such as microglia activation, p53-mediated apoptosis, inflammatory response, and free radical reactions [8, 11, 41, 42].

Quantification of Brn3a positive RGCs showed that administration of exogenous H_2_S correlated positively with RGC survival improvement in acute IOP elevation. The mass spectrometric-assisted proteomics analysis of the retinal tissue demonstrated that administration of H_2_S also further downregulated SNCB.

We may suggest that downregulating SNCB partly contributes to the neuroprotection by H_2_S under glaucomatous condition. The extent to which internal mechanism and/or inflammatory factors, signaling pathways, or the disruption of vascular function participate in the process is to be elucidated.

## 5. Conclusion

In conclusion, our results indicate that SNCB can transform from a neuroprotective to a neurodegenerative molecule. In physiological process, SNCB is neuroprotective; its level and the function of its self-regulation decreases with aging, which increases RGC's susceptibility to glaucomatous assaults. In pathological conditions, SNCB is neurotoxic; downregulation of SNCB is a self-protective mechanism, which presents from the beginning of IOP elevation, and the exhaustion of its functional reserve leads to irreversible neurodegeneration. Furthermore, increasing endogenous H_2_S is effective to downregulate SNCB and improve RGC survival against acute IOP elevation. Further detailed and in-depth investigation is required for comprehension of the roles of SNCB and H_2_S in glaucoma.

## Data Availability

The datasets generated during and/or analyzed during the current study are available from the corresponding author upon reasonable request.
